# NutriSteppe-AI: Development, Architecture, and Explainable Design of a Large Language Model–Driven Chatbot for Personalized Health Menu Generation

**DOI:** 10.3390/nu18142228

**Published:** 2026-07-09

**Authors:** Akkumis Salkhanova, Elnura Nabigazinova, Aliya Kaldybay, Ayaulym Omirbekova, Madina Sabit, Laura Baikonsova, Raushan Yergeshbayeva, Asyl Knyazbay, Timur Chuiko, Irina Yermakova, Aisulu Bekzhanova, Gulnara Tyulebekova, Danagul Niyetkaliyeva, Nursaya Serikova, Almaz Sharman

**Affiliations:** 1 Kazakh Academy of Nutrition, 66 Klochkov Street, Almaty 050008, Kazakhstan; asalkhanova@academypm.org (A.S.); elnura@academynutrition.kz (E.N.); akaldybai@academypm.org (A.K.); a.omirbekova@academypm.org (A.O.); m.sabit@academypm.org (M.S.); asyl.k@academypm.org (A.K.); 2 South Kazakhstan Medical Academy, 1 Al-Farabi Square, Shymkent 160019, Kazakhstan; laura_omarovna@mail.ru; 3 School of Public Health, Kazakh National Medical University, 94 Tole bi Street, Almaty 050012, Kazakhstan; raushanbogdanovna21@gmail.com; 4 School of Information Technology and Applied Mathematics, SDU University, 1/1 Abylai Khan Street, Kaskelen 040900, Kazakhstan; ttimkoni2005@gmail.com (T.C.); dananiyetka@gmail.com (D.N.); nursayanurzhankyzy07@gmail.com (N.S.); 5 Kazakhstan Academy of Preventive Medicine, 66 Klochkov Street, Almaty 050008, Kazakhstan; yermakova@academypm.org (I.Y.); bekzhanova@academypm.org (A.B.); tyulebekova@academypm.org (G.T.)

**Keywords:** artificial intelligence, large language models, nutrition, personalized diet, chatbots, clinical decision support systems, digital health, explainable AI, linear programming optimization, food databases

## Abstract

**Background/Objectives**: Suboptimal dietary patterns are among the leading modifiable contributors to global morbidity and mortality, particularly in cardiovascular disease, type 2 diabetes mellitus (T2DM), obesity, metabolic syndrome, and hypertension. Digital nutrition platforms have emerged to improve adherence to evidence-based dietary strategies; however, many systems lack structured optimization, processing-aware nutrient profiling, and explainable artificial intelligence (AI) mechanisms. The integration of large language models (LLMs) into digital health introduces conversational personalization but also risks hallucination and unsafe outputs without constraint enforcement. This study aimed to describe the system development, architecture, database infrastructure, optimization algorithms, explainability enforcement, and digital health implications of NutriSteppe-AI, a chatbot-first LLM-driven system for personalized health menu generation constrained by deterministic nutrient logic and processing-aware scoring. **Methods**: NutriSteppe-AI integrates: (1) a multi-source structured nutrient database of 20,000 food products with up to 130 tracked nutrients; (2) energy requirement estimation using the revised Harris-Benedict equation; (3) linear programming-based multi-objective optimization; (4) a Healthy Food Index (HFI; 0.5–5.0 scale) incorporating NOVA processing classification penalties; (5) traffic-light nutrient gating; and (6) a constrained LLM orchestration layer governed by structured API contracts. Algorithmic validation was performed using 10,000 simulated user profiles spanning diverse age, anthropometric, activity, dietary exclusion, and budget parameters. **Results**: The system achieved 96.8% full constraint satisfaction with macronutrient mean absolute errors of 11.60% (energy), 18.86% (protein), 16.26% (fat), and 20.91% (carbohydrates). Incorporating NOVA processing penalties reduced ultra-processed food HFI scores by 0.73 points (*p* < 0.001). Median optimized menu HFI improved from 3.6 to 4.3. Median system latency was 1.8 s. Explainability validation confirmed 100% deterministic alignment with zero hallucinated numeric claims. **Conclusions**: NutriSteppe-AI demonstrates that LLM-driven nutrition chatbots can achieve deterministic, explainable, and clinically aligned performance when governed by structured optimization, processing-aware scoring, and explainability enforcement. This architecture provides scalable digital health infrastructure for cardiometabolic disease prevention in diverse populations.

## 1. Introduction

### 1.1. Global Burden of Dietary Risk

Dietary risk factors constitute a leading contributor to global disease burden. The Global Burden of Disease Study estimated that suboptimal dietary patterns account for millions of deaths annually, primarily through cardiovascular and metabolic pathways [1,2]. Dietary strategies including the Dietary Approaches to Stop Hypertension (DASH) and Mediterranean patterns have demonstrated significant reductions in blood pressure, lipid abnormalities, glycemic dysregulation, and major cardiovascular events [3,4,5,6]. Nutritional modification remains foundational to the management of type 2 diabetes mellitus (T2DM) [7,8], obesity [9,10], and metabolic syndrome [11,12].

Despite robust evidence supporting dietary intervention, population-level adherence remains limited [8]. Digital health interventions have been explored as scalable tools to improve dietary behavior change [13,14,15,16], incorporating established behavioral science frameworks such as the transtheoretical model of behavior change and the behavior change wheel [17,18].

### 1.2. Nutrient Profiling and Food Processing Classification

Nutrient profiling systems—including Health Star Ratings and traffic-light labeling—were developed to guide consumers toward healthier food choices [19,20]. However, evidence indicates that ultra-processed foods may receive disproportionately favorable ratings under conventional profiling frameworks that do not account for the degree of industrial processing [21,22,23,24,25]. The NOVA food classification system addresses this limitation by categorizing foods according to their extent and purpose of processing [26,27,28,29].

Epidemiological evidence consistently links high ultra-processed food consumption to elevated risks of cancer, cardiovascular disease, and metabolic dysfunction [27,28]. Incorporating processing-adjusted penalties into nutrient scoring systems therefore improves alignment with public health goals and epidemiological findings [25,26].

### 1.3. Artificial Intelligence and Large Language Models in Healthcare and Nutrition

Artificial intelligence (AI) is increasingly integrated into clinical decision support, diagnostic imaging, and health behavior systems [15]. Large language models (LLMs) demonstrate advanced natural language reasoning and generation capabilities; however, they carry inherent risks of hallucination and non-deterministic outputs [30,31,32,33]. In healthcare contexts, these limitations necessitate robust governance frameworks emphasizing explainability, auditability, and clinical safety [34,35,36,37].

Recent evidence has raised important concerns regarding the reliability and safety of generative AI systems in the context of nutrition counseling and dietary guidance. In one study, five widely used chatbot platforms were tasked with generating three-day weight-loss meal plans for fictional adolescents with overweight or obesity. Compared with plans developed by a registered dietitian, the AI-generated menus contained substantially lower caloric content, averaging approximately 700 fewer kilocalories per day. The authors cautioned that sustained adherence to such restrictive dietary recommendations could increase the risk of malnutrition, impaired growth, and disordered eating behaviors among adolescents [38].

Similarly, another investigation evaluating chatbot responses to health-related queries found considerable shortcomings in nutrition guidance. Among 50 nutrition-related responses assessed, 72% were rated as ineffective or potentially harmful if implemented in practice [39]. These findings underscore the need for careful validation, expert oversight, and evidence-based safeguards when deploying AI systems in nutrition and public health settings.

Explainable AI methods such as SHAP (Shapley Additive Explanations) and LIME (Local Interpretable Model-agnostic Explanations) provide mechanisms for transparent model reasoning [35,36]. Digital health deployment frameworks emphasize structured decision support, human–AI partnership, and clinician-in-the-loop governance [40,41,42].

The current landscape of AI-driven dietary recommendation systems spans rule-based and collaborative-filtering nutrition recommenders, knowledge-based meal planners (e.g., the PROTEIN AI advisor [43]), deep-generative recommenders [44], and recent general-purpose LLM/GPT meal planners [38,39,45]. Across these systems, three recurrent limitations persist: (i) the absence of formal mathematical optimization guaranteeing simultaneous satisfaction of energy, macronutrient, and safety constraints, as most rely on heuristic substitution or unconstrained generation; (ii) the absence of processing-aware nutrient profiling, so that ultra-processed foods can receive favorable scores under conventional indices; and (iii) the absence of explainability and governance mechanisms to bound LLM hallucination in safety-critical nutritional output. NutriSteppe-AI advances beyond this state of the art by unifying all three within a single hybrid deterministic–generative architecture.

SHAP and LIME are referenced here only as established post hoc explainability methods for opaque models; they are not used in NutriSteppe-AI. The system instead provides intrinsic, deterministic, white-box explainability through an exact factor-contribution mechanism (Section 2.7), so post hoc approximation methods such as SHAP/LIME are neither required nor appropriate, because the contributions are exact rather than estimated.

### 1.4. Study Objective

NutriSteppe-AI was developed to integrate deterministic nutrient optimization, processing-aware scoring, traffic-light nutrient gating, LLM-driven conversational interface, and systematic explainability enforcement into a unified digital health platform. This manuscript describes the system architecture, database integration, optimization framework, validation methodology, and digital health implications of this approach.

The objective of this study was to develop and algorithmically validate a chatbot-first, LLM-driven menu-generation system in which all nutritional computation is performed by a deterministic, constraint-satisfying optimizer and the LLM is structurally confined to conversational interpretation and explanation, thereby delivering personalized, processing-aware, and fully traceable dietary recommendations.

## 2. Materials and Methods

### 2.1. Study Design and System Development Framework

**Figure 1:**
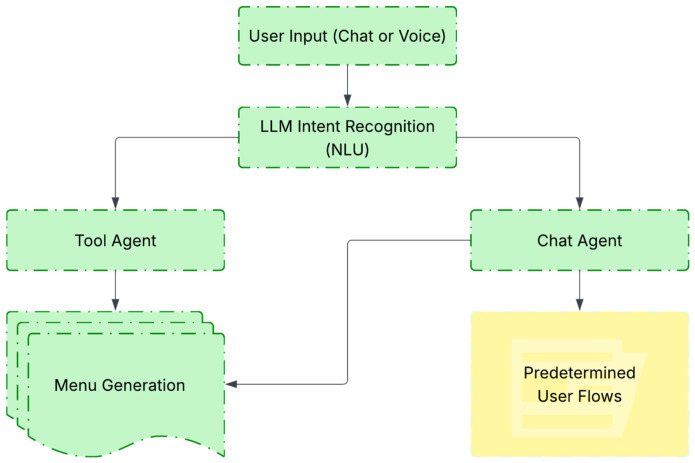
Hybrid deterministic–generative architecture of NutriSteppe-AI. User input is processed by LLM intent recognition (NLU), which routes to either a Tool Agent (triggering menu generation via deterministic optimization) or a Chat Agent (handling predetermined conversational user flows).

NutriSteppe-AI was developed through iterative agile cycles organized into three sequential phases: (1) Foundational Architecture Design, encompassing database construction, energy estimation, and constraint specification; (2) Optimization and Scoring Integration, including HFI formulation, NOVA penalty calibration, traffic-light gating, and linear programming solver implementation; and (3) LLM Orchestration and Explainability Enforcement, comprising API contract design, intent classification, structured output generation, and explanation validation. The system follows a hybrid deterministic–generative computational model in which all nutritional calculations are performed deterministically prior to LLM text generation (see Figure 1).

This study reports system development and algorithmic validation; no human participants were enrolled, and no patient data were used. Ethical review was not required under applicable institutional guidelines for computational system development.

Python 3.12 (Python Software Foundation, Wilmington, DE, USA), FastAPI 0.115.6 (FastAPI, Sebastián Ramírez, Mexico), and TypeScript 5 (Microsoft Corporation, Redmond, WA, USA) were used to develop the platform’s backend infrastructure. React 19 (Meta Platforms, Inc., Menlo Park, CA, USA) was used to create the front-end application. PostgreSQL 16 (PostgreSQL Global Development Group, USA) was used for data management, while Docker Engine 28.5.2 (Docker Inc., Palo Alto, CA, USA) was used for application deployment and containerisation. Visual Studio Code 1.102 (Microsoft Corporation, Redmond, WA, USA) was used for software development. GPT-5.4 nano (OpenAI, San Francisco, CA, USA) included large language model (LLM) features.

### 2.2. Database Architecture

#### 2.2.1. Multi-Source Food Database

The NutriSteppe nutrient database contains approximately 20,000 standardized food items with up to 130 tracked nutritional parameters per item. Of these, a validated core subset of 6916 foods and dishes was used for menu generation and nutrient calculations. This subset was compiled from the following reference databases: the German Nutrient Database (BLS), McCance and Widdowson’s Composition of Foods Integrated Dataset (CoFID), the Skurikhin Central Asian Nutrient Reference Database, USDA FoodData Central, and the Kazakhstani Food Composition Database.

The Kazakhstani Database refers to food-composition data compiled and maintained by the Kazakh Academy of Nutrition, comprising nationally consumed composite dishes and products whose values are derived from standardized recipe-based Technical Cards (“tekhnologicheskie karty”) and laboratory composition analysis. It is distinct from the Skurikhin Central Asian Reference Database, which is listed separately.

The database encompasses 6916 foods and dishes, distributed across 18 categories: meat (*n* = 1390), bread and bakery products (*n* = 1299), fish (*n* = 749), garnish preparations (*n* = 536), fruits and vegetables (*n* = 578), snacks (*n* = 327), desserts (*n* = 476), soups (*n* = 340), salads (*n* = 280), porridges (*n* = 53), beverages (*n* = 296), dairy products (*n* = 170), eggs (*n* = 114), cheeses (*n* = 49), cottage cheese preparations (*n* = 36), nuts and seeds (*n* = 52), sauces (*n* = 99), and appetizers (*n* = 72).

Each database entry contains structured fields including macronutrients, micronutrients, energy density, fiber content, sodium, saturated fat, added sugars, NOVA processing classification, allergen flags, seasonality metadata, recipe linkage, and Technical Card (TechCard) integration identifiers. User-generated menu plans are stored in a relational ‘menu_history’ table, enabling longitudinal dietary pattern tracking and personalized menu retrieval.

Added-sugar values have mixed provenance across the multi-source database. For reference-database items (BLS, CoFID, USDA), added values are taken as the analytical or database-reported values where available. For composite dishes and TechCard items, added sugars are computed from recipe composition as the sum of sugars contributed by discretionary sugar-containing ingredients, excluding intrinsic sugars from whole fruits and milk. For branded/ultra-processed items, added sugars are derived from manufacturer label declarations.

To reflect Kazakhstan’s food landscape, the Kazakh Academy of Nutrition’s nutritionists categorized all 20,000 dishes and products into five availability groups: items available daily across Kazakhstan, restaurant-specific dishes and products, exotic ingredients and meals, ultra-processed foods, and unclassified items. For menu generation training, the system uses two availability types: daily available items (*n* = 4081) and restaurant dishes and products (*n* = 2828).

**Figure 2:**
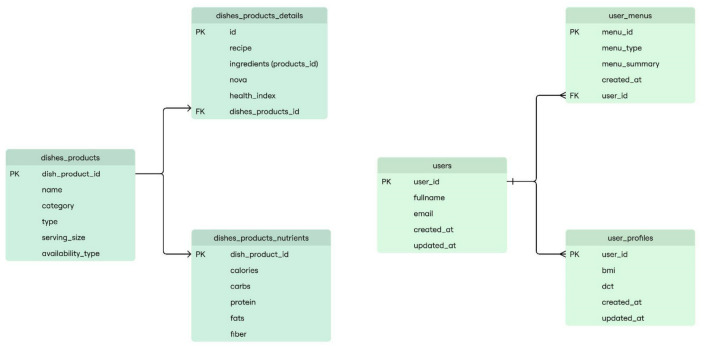
Relational database schema of NutriSteppe-AI.

The relational database schema of NutriSteppe-AI is presented in Figure 2. The schema links the core dishes and products table (dish_product_id, name, category, type, serving_size, availability_type) to dish and product details (id, recipe, ingredients, health_index, nova) and dish and product nutrients (macronutrient and energy fields). User data are stored across the users, user_profiles (BMI, daily caloric targets), and user_menus (generated menu history) tables, with foreign key relationships enabling personalized menu retrieval.

#### 2.2.2. Energy Requirement Estimation

Basal Energy Expenditure (BEE) is calculated using the revised Harris–Benedict equation [46]

Males: BEE = 88.36 + (13.4 × W) + (4.8 × H) − (5.7 × A) Females: BEE = 447.6 + (9.2 × W) + (3.1 × H) − (4.3 × A)(1)

where W = body weight (kg), H = height (cm), and A = age (years). Total Energy Expenditure (TEE) is derived as TEE = BEE × ActivityFactor ± GoalAdjustment, with activity factors ranging from 1.2 (sedentary) to 1.9 (very active). Macronutrient target ranges follow WHO and Institute of Medicine reference values [47,48,49].

### 2.3. Healthy Food Index

The Healthy Food Index (HFI) is scored on a 0.5 to 5.0 scale in 0.5-point increments. The composite score integrates six positive contributors—fiber density, fruit/vegetable/nut/legume (FVNL) content, protein adequacy, unsaturated fat ratio, energy density efficiency, and micronutrient density—and four penalty components: added sugar load, sodium content, saturated fat proportion, and NOVA processing classification. The NOVA-derived processing penalty ensures that ultra-processed foods (NOVA Group 4) receive systematically lower HFI scores, operationalizing the alignment of the scoring system with current epidemiological evidence [11,12,27,28].

### 2.4. Traffic-Light Nutrient Gating

Per 100 g nutrient thresholds classify food items for sugar, salt, fat and saturated fat into three traffic-light categories (green/yellow/red) following established UK Food Standards Agency criteria [19,20,50]. These categorical designations function as upstream gating filters applied before candidate food ranking in the optimization pipeline, ensuring that items with red-level concentrations of high-risk nutrients are deprioritized or excluded under configurable dietary protocols.

### 2.5. Linear Programming Optimization

Menu optimization employs a Simplex-based linear programming solver [51,52]. The multi-objective function is defined as

Minimize J = λ_1_|E_target_ − E_menu_| + λ_2_(1 − HFI_avg_) + λ_3_Cost (2)

Here, J is the objective function; E_target_ is the required energy intake; E_menu_ is the total energy provided by the menu; HFI_avg_ is the average Healthy Food Index; Cost is the total menu cost; and λ_1_, λ_2_, and λ_3_ are weighting coefficients controlling the importance of energy balance, nutritional quality, and cost, respectively.

Subject to the following constraints: macronutrient proportions within WHO-recommended bounds; minimum dietary fiber threshold (≥25 g/day); sodium upper limit (≤2000 mg/day per WHO guidance [49]); saturated fat cap (≤10% of total energy); complete allergen exclusions per user profile; dietary pattern adherence (DASH, Mediterranean, ketogenic, or custom); seasonality availability constraints; minimum HFI threshold per meal; and traffic-light gating compliance.

Palatability and variety are handled through structural constraints through a combination of structural optimization constraints and user-centered personalization rather than the objective function alone. The optimizer assembles menus by meal slot (breakfast, lunch, dinner, snacks), and each slot draws only from category sets appropriate to it (for example, desserts and soups are not eligible for the breakfast slot), which prevents implausible combinations. Variety is enforced by a no-repeat constraint that limits how often the same dish or category may recur within a defined window (e.g., across the days of a weekly plan), and by category-balance constraints that ensure a spread across food groups. Menu generation also incorporates user-specific preferences and medical requirements. Allergies, health conditions, and dietary restrictions are treated as mandatory constraints, while disliked foods are excluded and preferred foods are prioritized whenever compatible with the nutritional requirements. In addition, NutriSteppe-AI provides a shuffle function that replaces a selected dish with a nutritionally equivalent alternative from the same food category while preserving the overall nutritional profile of the meal. Users may also request substitutions in natural language, which are interpreted by the LLM and mapped to suitable alternatives that satisfy the same dietary constraints, thereby improving both menu acceptability and long-term adherence.

The database tracks up to 130 nutritional parameters, but not all are imposed as optimization constraints. The linear program enforces a defined set of hard and soft constraints on the nutrients with established reference values most relevant to cardiometabolic risk (energy, the three macronutrients within WHO bounds, fiber ≥ 25 g/day, sodium ≤ 2000 mg/day, saturated fat ≤ 10% of energy). The remaining micronutrients are tracked and reported, but are not each individually bounded—a deliberate design choice that keeps the problem well-posed and tractable.

### 2.6. LLM Orchestration Layer

The LLM layer performs four functions within the system: intent recognition (classifying user queries into canonical intents: PLAN_DAY, PLAN_WEEK, SWAP_ITEM, BUILD_BASKET, EXPLAIN_SCORE, and ADJUST_PREFERENCE); structured API endpoint invocation via POST/v1/menu/generate; natural language explanation synthesis from structured JSON output; and user preference adaptation through conversational context updating. The LLM does not perform any nutritional calculations. It consumes structured JSON output from the deterministic optimization engine, which includes: energy_kcal_planned, avg_health_index, traffic_light_daily summary, individual meal objects with nutrient breakdowns, swap candidate lists, shopping list, and optimization metadata. This architectural separation ensures that all nutritional values and constraint-satisfaction claims are generated by verified deterministic processes.

The deployed system uses GPT-5.4 Nano exclusively for intent recognition and natural language interaction. All nutritional calculations, menu optimization, Health Food Index (HFI) scoring, and constraint validation are performed by the deterministic optimization engine. The LLM is not involved in any computational or decision-making processes affecting nutritional outputs.

The architecture is designed to fail safely. Intent classification returns a confidence score; below a threshold, the system does not trigger menu generation but instead issues a clarification prompt or routes the user to the Chat Agent’s predefined flows. Menu generation is invoked only when a recognized intent yields a fully validated structured request payload. Unsupported or out-of-scope requests are routed to predefined conversational handling rather than free-form generation. If the optimizer subsequently returns an infeasible problem (the 3.2% high-restriction cases), a constraint-relaxation hierarchy is applied and the relaxation is disclosed to the user.

The menu-generation endpoint (POST/v1/menu/generate) accepts a typed request object (sex, age, weight, height, activity factor, goal, dietary pattern, allergen exclusions, budget) and returns a typed response object containing energy_kcal_planned, avg_health_index, traffic_light_daily, per-meal nutrient breakdowns, swap-candidate lists, shopping list, and optimization metadata. Validation rules include range checks on anthropometrics, enumerated intents and dietary patterns, and mandatory allergen-exclusion enforcement before any candidate ranking.

This section is expanded in three ways. First, the prompt structures are described and included: the intent-classification prompt (mapping utterances to the canonical intents) and the explanation-synthesis prompt, whose system instruction constrains the LLM to consume only the deterministic JSON and explicitly forbids it from computing or altering any nutritional value. Second, a complete worked example traces one request end-to-end (below). Third, a generalizability note is added: the deterministic-core/generative-surface separation transfers to other cuisines (by swapping the food database) and to other constrained-recommendation domains (by replacing the objective and constraints) without changing the orchestration logic.

**Worked example (to be set as a boxed panel). User utterance:** “Make me a one-day menu—45-year-old man, 92 kg, 178 cm, moderately active, I want to lose weight, no fish or seafood, keep it affordable.”

**Intent recognition (LLM/NLU):** intent = PLAN_DAY; extracted entities → {sex: male, age: 45, weight_kg: 92, height_cm: 178, activity_factor: 1.55, goal: weight_loss, exclusions: [fish/seafood], budget_level: low}.

**Deterministic computation:** BEE (revised Harris–Benedict, male) = 88.36 + 13.4 × 92 + 4.8 × 178 − 5.7 × 45 ≈ 1919 kcal; TEE = 1919 × 1.55 ≈ 2975 kcal; with a −500 kcal weight-loss adjustment, energy target ≈ 2470 kcal. The LP solver then assembles a fish-free, low-cost menu satisfying all macronutrient, fiber, sodium, saturated-fat, and traffic-light constraints.

**Structured JSON (abridged):** {energy_kcal_planned: 2470, avg_health_index: 4.3, traffic_light_daily: {sugar: green, salt: yellow, sat_fat: green}, meals: […], shopping_list: […]}.

**LLM explanation (constrained to the JSON):** “Here is your plan for today at about 2470 kcal, consistent with a ~500 kcal/day deficit for gradual weight loss. The average Healthy Food Index is 4.3 out of 5.0. Fish and seafood are fully excluded. Salt sits in the yellow band, mainly from the lunch soup—tap ‘swap’ to bring it to green.” Every number in this sentence is present in the structured payload.

The application of LLM-based intent recognition is motivated by empirical research demonstrating that LLMs offer uniform benefits in zero-shot intent classification scenarios, where user utterances are mapped to pre-defined intent classes, efficiently avoiding task-specific fine-tuning [53].

**Figure 3:**
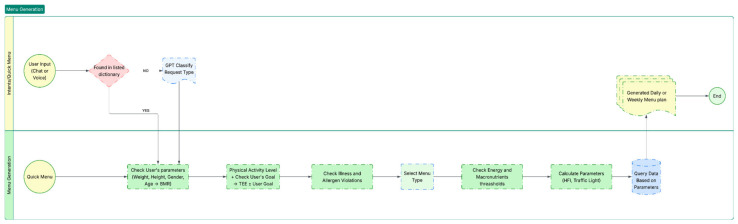
End-to-end menu generation workflow of NutriSteppe-AI.

The end-to-end menu generation workflow of NutriSteppe-AI is illustrated in Figure 3. User input (chat or voice) or a Quick Menu request triggers a UI routing decision. The menu generation pipeline then sequentially processes user anthropometric parameters (weight, height, gender, age → BMR estimation), computes TEE with adjustments for physical activity level and dietary goals, evaluates medical conditions and allergen constraints, selects the appropriate menu type, applies energy and macronutrient thresholds, calculates the HFI and traffic-light nutrition scores, and queries the food database to generate a personalized daily or weekly menu plan.

### 2.7. Explainability Enforcement

The explainability pipeline comprises four sequential stages: (1) Scoring Engine generates factor-level contribution weights for each HFI component; (2) Factor Contribution Table maps each nutrient or processing penalty to its numerical contribution; (3) Explanation Template selects the appropriate natural language template for each factor tier; and (4) LLM Surface Output renders the explanation in conversational language anchored to the template. Each explanation token is traceable to a specific scoring factor. Validation confirmed 100% deterministic alignment, zero hallucinated numeric claims, and complete nutrient contribution traceability across all simulated profiles.

The HFI is a deterministic weighted composite. Each of the three positive contributors (fiber density, FVNL content, protein adequacy) and five penalty components (energy-density, added-sugar load, sodium, saturated-fat proportion, NOVA processing class) is mapped to a normalized sub-score; each sub-score is multiplied by a fixed weight, and the weighted sub-scores are summed (positives added, penalties subtracted) and mapped onto the 0.5–5.0 scale. The factor-level contribution reported in the explainability layer is precisely each component’s signed weighted sub-score, so the contributions sum to the final HFI by construction.

The zero-hallucination and complete-traceability results follow from the architecture and were verified by an automated audit. Because every numeric value originates in the deterministic optimizer and is injected into fixed explanation templates, all 10,000 generated outputs were audited by programmatically extracting each numeric token in the rendered explanation and matching it against the values in the corresponding structured JSON payload; an output was counted as containing a hallucinated numeric claim if any rendered number did not exactly match a source value. For traceability, every factor and numeric value in each rendered explanation was matched against an entry in the Factor Contribution Table, and an output passed only if every referenced factor/number mapped to a source entry and no extraneous factor or numeric claim appeared. No mismatches were found (denominator = 10,000; pass rate 100%).

The proposed Health Food Index (HFI) adopts the nutrient profiling principles of the Health Star Rating (HSR) system developed by Australia and New Zealand. The HSR methodology evaluates packaged foods by assigning baseline points for energy, saturated fat, total sugars, and sodium, which are subsequently adjusted using positive components including fruit, vegetable, nut and legume (FVNL) content, dietary fiber, and protein where applicable. All calculations are performed per 100 g or 100 mL of product using the official HSR Calculator and Style Guide. In addition to nutrient profiling, the HFI also considers the degree of food processing according to the NOVA classification, distinguishing foods into four groups based on the nature, extent, and purpose of industrial processing [26,54].

### 2.8. Algorithmic Validation

Algorithmic validation was conducted using 10,000 simulated user profiles generated by Monte Carlo sampling across the following parameter distributions: age 18–75 years; BMI 18.0–40.0 kg/m^2^; multiple physical activity levels; diverse dietary exclusion combinations; and household budget variability. Simulated profiles included common clinical presentations relevant to cardiometabolic prevention, including hypertension, dyslipidemia, T2DM risk, and obesity.

Primary validation metrics included: (1) constraint satisfaction rate; (2) macronutrient mean absolute error (MAE) against algorithmic targets; (3) allergen violation rate; (4) HFI distributional shift between random baseline and optimized output; (5) explainability consistency; and (6) end-to-end system latency.

## 3. Results

### 3.1. Constraint Adherence

Full constraint satisfaction was achieved in 96.8% of simulated profiles. Minor constraint relaxations occurred in 3.2% of profiles, exclusively under high-restriction conditions combining multiple simultaneous allergen exclusions, strict budget limits, and narrow seasonality windows. The allergen violation rate was 0%, confirming absolute safety constraint enforcement across all 10,000 simulations.

### 3.2. Nutritional Accuracy

**Figure 4:**
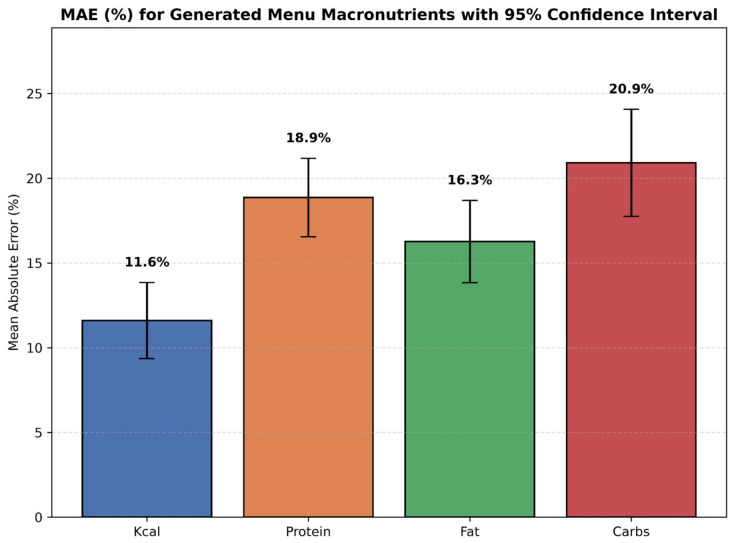
Mean absolute error (MAE, %) with 95% Confidence Interval for macronutrient targets across 10,000 simulated user profiles generated by the NutriSteppe-AI optimization engine.

Macronutrient mean absolute errors (MAE) against optimization targets were as follows: energy 11.60%, protein 18.86%, fat 16.26%, and carbohydrates 20.91%. Mean optimized menu HFI was 3.8 across all simulated profiles. Table 1 presents the full macronutrient accuracy summary, and Figure 4 illustrates the MAE with CI distribution across macronutrient categories.

These values are in line with optimal benchmarks for AI-based nutrition accuracy, where GPT-based systems with full meal context achieved a mean absolute percentage error (MAPE) of 13.7% for carbohydrate estimation, whereas systems operating under moderate data conditions reported errors of 18.1% [45]. One identified limitation of AI-generated diet plans is macronutrient balance and distribution, with some systems producing caloric deviations exceeding 20% in over half of generated plans [55]. Adding an optimization layer to AI-based diet generation has been shown to significantly improve macronutrient accuracy by adjusting meal quantities toward target energy and nutrient values [44]. The standard evaluation metrics for AI nutrition generation systems are MAE and MAPE over energy, protein, fat, and carbohydrates [43,44,45]. Experiments on 3000 to 84,000 virtual user profiles can be considered a rigorous methodology to demonstrate the accuracy of AI-based dietary recommendation systems [43,44].

### 3.3. Processing-Aware Discrimination

NOVA classification-based processing penalties demonstrated statistically significant discriminatory validity. The mean HFI score for ultra-processed foods (NOVA Group 4) decreased by 0.73 points following penalty application (*p* < 0.001), consistent with the intended directional effect of the scoring algorithm. This magnitude of discrimination enables the system to meaningfully distinguish ultra-processed items from minimally processed alternatives within the same food category.

### 3.4. Menu Quality Improvement

Median HFI of randomly assembled menus without optimization was 3.6 (pre-intervention reference). Following linear programming optimization, median HFI improved to 4.3, representing a clinically meaningful shift of 0.7 HFI points. The proportion of optimized menus achieving HFI ≥ 4.0 was substantially higher than baseline random assembly, reflecting the systematic effect of multi-objective constraint-aware optimization.

On the 0.5–5.0 HFI scale, a 0.7-point gain corresponds to roughly 14% of the full range and moves the median menu across the HFI ≥ 4.0 threshold used to denote high dietary quality; in practical terms, the optimization shifts the bulk of the distribution from “moderate” into the “high-quality” band, driven by greater fiber and FVNL content and reduced ultra-processed contribution. The HFI is an internally constructed composite built from externally validated components (NOVA classification, FSA thresholds, WHO/IOM reference values), but the composite index itself has not yet been independently validated against established indices or health outcomes.

### 3.5. System Performance

Median end-to-end system latency was 1.8 s, with a 95th-percentile latency of 2.4 s. Linear programming optimization runtime was below 200 ms in 97.3% of simulated queries. These performance characteristics are consistent with real-time interactive application requirements.

### 3.6. Explainability Consistency

Explainability validation confirmed 100% alignment between numerical scoring factor contributions and generated natural language explanations. No hallucinated numeric claims were identified across any of the 10,000 simulated profiles. Every generated explanation was fully traceable to a specific factor in the HFI scoring engine, satisfying the system’s explainability enforcement criteria.

## 4. Discussion

### 4.1. Principal Findings

NutriSteppe-AI demonstrates that LLM-driven nutrition chatbots can be made deterministic, clinically aligned, and fully explainable when governed by structured optimization, processing-aware scoring, and architectural separation between deterministic computation and generative language output. The system achieved 96.8% full constraint satisfaction across a diverse simulated population, eliminated allergen violations, and produced significant HFI discrimination between ultra-processed and minimally processed foods consistent with current epidemiological evidence [27,28]. Explainability enforcement achieved complete traceability across all simulated outputs, addressing a fundamental governance requirement for AI systems deployed in clinical and public health contexts [34,35,36,37].

### 4.2. Comparison with Prior Digital Nutrition Systems

Existing digital nutrition platforms and dietary mobile applications largely lack structured mathematical optimization, processing-aware nutrient profiling, and formal explainability mechanisms [13,14,15,16]. Many commercial systems rely on heuristic food substitution or user-driven filtering without constraint enforcement. Prior AI-driven dietary recommendation systems have demonstrated value in improving nutritional knowledge and short-term adherence [15,16]; however, governance frameworks addressing LLM hallucination, safety constraint enforcement, and explanation traceability have not been systematically implemented in published digital nutrition systems.

NutriSteppe-AI advances this field by combining linear programming-based multi-objective optimization—a method with historical precedent in clinical dietetics [51,52]—with a constrained LLM orchestration layer that limits generative outputs to pre-computed, verified nutritional data. This architecture directly addresses the hallucination risk identified in LLM healthcare deployment [30,31,32,33] through structural rather than prompt-based constraint mechanisms.

### 4.3. Clinical and Public Health Implications

The clinical alignment of NutriSteppe-AI is supported across multiple cardiometabolic disease domains. In cardiovascular disease prevention, the system enforces saturated fat and sodium gating consistent with Mediterranean and DASH dietary principles and WHO guidelines [4,5,6,49,56]. In hypertension management, sodium restriction is operationalized as a hard constraint rather than a recommendation, ensuring dietary outputs meet clinical targets [4,49]. For T2DM prevention and management, optimized menus support evidence-based dietary modification strategies [7,57,58]. In obesity and metabolic syndrome management, the energy balance optimization and HFI maximization framework aligns with lifestyle modification principles [9,10,11,12].

The Central Asian epidemiological context—characterized by high burdens of cardiovascular disease, T2DM, and obesity alongside limited access to personalized dietary counseling—makes scalable AI-driven nutrition systems particularly relevant for regional public health priorities.

### 4.4. Strengths

Key methodological strengths include: (1) a multi-source validated nutrient database with over 20,000 food products and culturally adapted regional coverage; (2) mathematically rigorous deterministic computation decoupled from LLM text generation; (3) NOVA processing-aware scoring aligned with epidemiological evidence; (4) formal explainability enforcement with complete factor traceability; (5) structured API governance preventing unsafe or hallucinated nutritional outputs; and (6) large-scale algorithmic validation across 10,000 diverse simulated profiles.

### 4.5. Limitations

Several limitations warrant acknowledgment. First, the validation reported here is computational and algorithmic; no prospective clinical trial evaluating health outcomes in real-world users has yet been conducted. Second, HFI scoring of beverage FVNL content has inherent measurement limitations due to variable bioavailability and concentration effects. Third, the current cultural localization reflects Central Asian and Eastern European food patterns; expansion to additional cultural contexts requires systematic database augmentation. Fourth, long-term user adherence to AI-generated dietary recommendations has not been empirically assessed in this population.

Fifth, comprehensive per-micronutrient adequacy (e.g., iron, calcium, vitamin D, B12, folate) is not yet individually constrained in the optimizer; these nutrients are tracked and reported but are not each bounded, and individual constraints are planned for future versions. Sixth, the zero-hallucination guarantee is strongest for numeric content; whether non-numeric hallucinations (unsupported qualitative claims, invented foods or constraints) were systematically assessed should be stated, as the present audit verifies exact numeric correspondence. Seventh, the Healthy Food Index, although built from externally validated components, has not yet been independently validated as a standalone composite index against established indices or health outcomes.

### 4.6. Future Directions

Priority future work includes: (1) prospective randomized controlled trials evaluating NutriSteppe-AI against standard care in cardiometabolic disease prevention; (2) integration with electronic medical record systems to enable clinician-facing dietary recommendation support; (3) wearable device and continuous glucose monitor data integration for real-time adaptive menu personalization; (4) microbiome-informed scoring refinement; (5) behavioral personalization loops incorporating longitudinal user interaction data; and (6) EMR-linked registry studies to evaluate population-level dietary quality improvements.

## 5. Conclusions

NutriSteppe-AI establishes a reproducible, explainable, and processing-aware LLM-driven architecture for personalized dietary menu generation. By integrating deterministic multi-objective linear programming optimization, NOVA-adjusted Healthy Food Index scoring, traffic-light nutrient gating, and a constrained LLM orchestration layer with systematic explainability enforcement, the platform provides scalable digital health infrastructure aligned with evidence-based cardiometabolic disease prevention. Algorithmic validation across 10,000 simulated profiles demonstrated robust constraint satisfaction, meaningful HFI improvement, zero allergen violations, and complete explanation traceability. Clinical validation trials are the necessary next step to demonstrate real-world health outcomes.

## Figures and Tables

**Table 1:** Macronutrient mean absolute error (MAE) and mean Healthy Food Index (HFI) score across 10,000 simulated validation profiles.

Nutritional Parameter	MAE (%)	SD%	95% CI	Clinical Interpretation
Energy (kcal)	11.60	11.40	[9.35, 13.84]	Within acceptable clinical tolerance
Protein (g)	18.86	11.74	[16.55, 21.17]	Within acceptable clinical tolerance
Fat (g)	16.26	12.31	[13.83, 18.70]	Within acceptable clinical tolerance
Carbohydrates (g)	20.91	16.03	[17.74, 24.06]	Within acceptable clinical tolerance
Mean HFI Score	3.8 (scale 0.5–5.0)			Above population reference median

## Data Availability

The algorithms and database schema described in this manuscript are available upon reasonable request to the corresponding author. The NutriSteppe nutrient database is maintained by the Kazakh Academy of Nutrition, Almaty, Kazakhstan.

## Abbreviations

The following abbreviations are used in this manuscript:

AI	Artificial Intelligence
API	Application Programming Interface
BEE	Basal Energy Expenditure
BLS	Bundeslebensmittelschlüssel (German Nutrient Database)
CoFID	Composition of Foods Integrated Dataset
CVD	Cardiovascular Disease
DASH	Dietary Approaches to Stop Hypertension
FVNL	Fruit, Vegetable, Nut, Legume
HFI	Healthy Food Index
HSR	Health Star Rating
JSON	JavaScript Object Notation
LIME	Local Interpretable Model-Agnostic Explanations
LLM	Large Language Model
MAE	Mean Absolute Error
MAPE	Mean Absolute Percentage Error
NLU	Natural Language Understanding
NOVA	Food Processing Classification System (Monteiro et al.)
SHAP	Shapley Additive Explanations
T2DM	Type 2 Diabetes Mellitus
TEE	Total Energy Expenditure
USDA	United States Department of Agriculture
